# Anthocyanins and Human Health—A Focus on Oxidative Stress, Inflammation and Disease

**DOI:** 10.3390/antiox9050366

**Published:** 2020-04-28

**Authors:** Hollie Speer, Nathan M. D’Cunha, Natalie I. Alexopoulos, Andrew J. McKune, Nenad Naumovski

**Affiliations:** 1 Faculty of Health, University of Canberra, Bruce, ACT 2617, Australia; hollie.speer@canberra.edu.au (H.S.); nathan.dcunha@canberra.edu.au (N.M.D.); andrew.mckune@canberra.edu.au (A.J.M.); 2 Faculty of Science and Technology, University of Canberra, Bruce, ACT 2617, Australia; 3 University of Canberra Research Institute for Sport and Exercise (UC-RISE), University of Canberra, Bruce, ACT 2617, Australia; 4 Chiron Organic Health, Wedderburn, VIC 3518, Australia; info@chironorganic.com.au; 5 Discipline of Biokinetics, Exercise and Leisure Sciences, School of Health Sciences, University of KwaZulu-Natal, Durban, KwaZulu-Natal 4000, South Africa

**Keywords:** anthocyanins, flavonoids, antioxidant activity, inflammation, disease, oxidative damage

## Abstract

Consumption of anthocyanins (ACNs), due to their antioxidant, anti-inflammatory and anti-apoptotic effects, has been proposed for the prevention and treatment of several different diseases and conditions. ACNs are recognized as one of the leading nutraceuticals for prolonging health benefits through the attenuation of oxidative stress, and inflammatory or age-related diseases. Increased consumption of ACNs has the potential to attenuate the damage ensuing from oxidative stress, inflammation, enhance cardiometabolic health, and delay symptoms in predisposed neuropathology. A myriad of evidence supports ACN consumption as complementary or standalone treatment strategies for non-communicable diseases (NCDs) including obesity, diabetes, cardiovascular disease (CVD), neurodegenerative diseases, as well as, more recently, for the modulation of gut bacteria and bone metabolism. While these findings indicate the beneficial effects of ACN consumption, their food sources differ vastly in ACN composition and thus potentially in their physiological effects. Consumption of foods high in ACNs can be recommended for their potential beneficial health effects due to their relatively easy and accessible addition to the everyday diet.

## 1. Introduction

Similar to most plant bioactive compounds, anthocyanins (ACNs) are found in a ‘natural’ abundance within the human diet and are proposed to induce positive effects on overall health [1,2]. Anthocyanins are water-soluble plant pigments that are prominent in the flesh, skin and roots of many coloured fruits and vegetables such as berries, plums and cherries [2]. Over the last decade, there has been an emphasis on researching the diet–disease relationship, together with the promotion of a nutrient-dense diet and exploration of ‘value-added’ food for their use as potential therapeutic strategies to minimize adverse health risks [2,3]. Relatively recent evidence has also emerged from clinical trials suggesting that the consumption of specific foods, beverages and nutraceuticals, such as those rich in ACNs, is associated with reduced risk for chronic, non-communicable diseases (NCDs) [2,4]. Non-communicable diseases comprise a ‘cluster’ of diseases including cancers, obesity, diabetes and cardiovascular disease (CVD), accounting for the majority of deaths in developed countries [2,4].

The ACN composition can vary between different sources and varieties of foods [5], depending on whether they are fresh, frozen or dried [6], with seasonability and environmental stress [7], as well as with food storage and preparation techniques (i.e., peeled, unpeeled) indicating that their presence might be concentrated in specific areas of fruits and vegetables such as the skin or flesh [5,8]. In 2002, the consumption of ACNs was estimated to be 12.5 mg/day/person in the United States, although it is anticipated that this number has risen due to the increased popularity of the so-called ‘superfood’ market for health and wellbeing [9,10,11]. Although widely distrubted in a variety of different coloured fruits and vegetables, common sources of ACNs include blueberries, blackberries, raspberries, and aronia fruits, while less common sources include black legumes and the purple prickly pear [12,13,14,15]. These common sources (Table 1) have concentrations ranging from 127 to 15,000 mg/kg, and the application of ACNs as a food source of treatment is frequently limited by their low bioavailability [12,14,16].

ACNs have also been proposed to attenuate reactive oxygen species (ROS) and reactive nitrogen species (RNS) in human, animal and *in vitro* studies [17,18,19,20]. The ROS are typically produced in the cytosol, mitochondria, endoplasmic reticulum and lysosomes, whereas RNS are produced primarily as a result of amino acid metabolism [21]. Both ROS and RNS are produced under normal circumstances by all aerobic cells and play an important part in cellular aging, as well as in pathologic inflammation and the progression of age-related NCDs [21,22]. A synergistic relationship between oxidative stress and NCDs has emerged as an important area of focus used for the identification of novel diagnostic biomarkers, as well as potential drug and therapeutic intervention strategies for the management and prevention of metabolic syndrome, diabetes, CVD and some neurodegenerative disorders [23].

The consumption of different ACNs in the form of foods, beverages and nutraceuticals has been investigated for the antioxidant effects they exhibit, as well as for their role in mediating inflammation, affecting body composition, cardiometabolic biomarkers, neurological pathology, gut microbiota, and a general delayed onset of disease [17,24,25,26]. However, there is an emerging need for an integrated focus on ACN consumption and their associated effects on commonly occurring NCDs across the lifespan, as well as recent evidence that they may enhance overall quality of life [4]. Therefore, this review will focus on recent human, animal and in vitro studies, assessing the consumption of ACNs and ACN-rich foods as potential therapeutics for mitigating oxidative damage, targeting inflammation and other pathways associated with the pathogenesis of chronic inflammatory or age-related NCDs.

## 2. Methods

Herein, we discuss the involvement of ACNs and the supportive roles they carry out in relation to oxidative damage, body composition, metabolic syndrome, as well as cardiovascular and neurological diseases. The scope of this review also extends to these factors contributing to a general delay in disease onset, as well as exploring more recent and emerging evidence.

A comprehensive database search was initially carried out in January 2020 and subsequently repeated in April 2020. Searches were conducted across CINAHL, PubMed, SCOPUS, and the Cochrane Library electronic databases. Searches were performed using a combination of the following terms and their derivations: ‘anthocyanin’, ‘oxidative stress’, ‘chronic inflammation’, ‘free radical’ and ‘disease’. Additionally, reference lists of relevant, previously published review articles were searched. Several reviews have focused on the effects of ACN consumption and the effects on overall health in a broad sense, therefore a comprehensive review focusing on these integrated pathways is needed. Thus, subtopics representing these specific integrated processes and diseases were selected based on their prevalence and significance to the research field.

## 3. Discussion of Findings

### 3.1. Oxidative Damage, ROS and RNS Production

**Figure 1:**
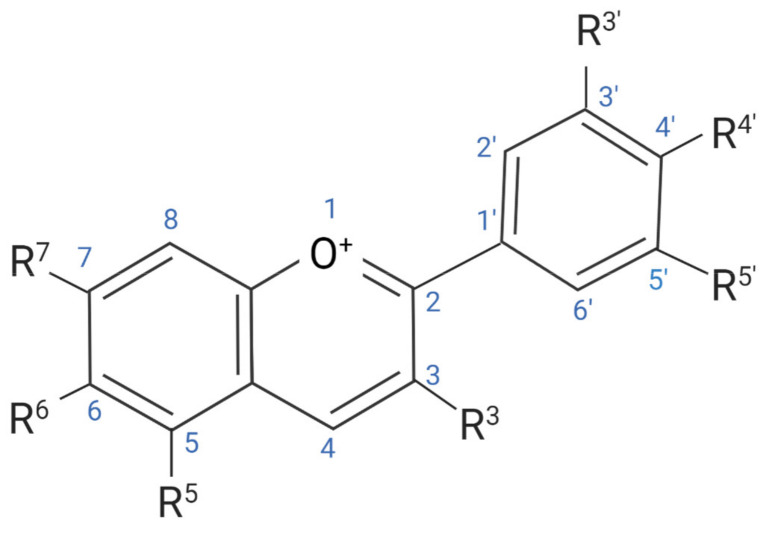
Basic chemical structure of anthocyanins.

Oxidative damage is proposed to play an important and causative role in the initiation, development and progression of several NCDs [22,27]. The damage ensued from ROS/RNS interactions has been associated with inflammation, lipid oxidation and several neurodegenerative disorders [28,29]. It is proposed that this mainly occurs due to the oxidative modification of major cellular macromolecules such as carbohydrates (CHOs), lipids, proteins, and more specifically, DNA [22]. With the ability to potentially scavenge free radicals and reduce oxidative stress, ACNs from a diet high in fruits and vegetables have been linked to slowing oxidative damage progression [30,31,32]. Endogenously, sources of ROS and RNS can occur via the nicotinamide adenine dinucleotide phosphate (NADPH) pathway, and when NADPH oxidase is activated, a large amount of oxygen (O_2_) and hydrogen peroxide (H_2_O_2_) are generated, causing extensive cellular proliferation [21]. If not suppressed, this can further lead to detrimental cellular changes and the initiation of many inflammatory and age-related diseases [21,33]. Dietary antioxidants are proposed to protect biological systems from free radical toxicity via different pathways, and as potent antioxidants, ACNs possess the ability to act as reducing agents in the electron-transfer reaction pathway, donating electrons to the free radicals with unpaired electrons [2,34]. It is essential to consider that the proposed antioxidant capabilities of ACNs may also be attributable to the non-ACN properties commonly found in most ACN-rich food sources such as Vitamin C [30]. However, it has been postulated that the number of hydroxyl groups present on the glycosylated B-ring structure (Figure 1) of ACNs is associated with their scavenging ability, and may directly influence their antioxidant capacity [2]. A study by Jian-Yun et al. (2015) compared the total antioxidative capability (T-AOC) of two ACN sources, the black bean peel extract and pomegranate peel extract, on oxidative stress-induced hyperglacemia in mice [35]. The black bean peel extract had slightly higher antioxidative capability (13 U/mL T-AOC) compared to that of the pomegranate peel extract (10 U/mL T-AOC) [35]. This may also suggest that the color intensity of ACN sources might aid in identifying higher antioxidant properties [36]. In a 2018 study by Ali et al. (2018), mice who received an ACN supplement derived from Korean black beans (12 mg/kg) for thirty days overcame ROS activation and had significantly reduced markers for oxidative stress and lipid peroxidation when compared with a saline vehicle control group [18]. This indicates a valuable consideration for the use of ACNs, specifically those with intense pigmentation, for overcoming damage ensued by oxidative stress. While most food sources contain varying levels of ACNs, it should also be considered how the food source may affect the scavenging abilities, and if the darker or more intense the pigment is—the higher the antioxidant capacity it possesses [36,37].

### 3.2. Body Composition

It is well accepted that body composition is an indicator of systemic nutritional and general health status, and can also provide valuable information for the diagnosis and treatment of several diseases [38]. According to the World Health Organisation (WHO), more than 1.9 billion adults were classified as overweight in 2016, with more than 650 million of these adults considered obese [39] and it is expected that this number is only going to increase. This highlights the growing need for novel interventions for the treatment of NCDs that are both manageable and maintainable [34,40,41]. Body composition is an important factor for the diagnosis of obesity along with other diseases, and measurements of body mass index (BMI), visceral fat percentage, and waist circumference in combination with inflammatory biomarkers such as adipokine/adipocytokine levels, are useful markers indicating the extent of the physiological burden [34]. There is growing evidence that increased visceral fat, adipokines and a state of obesity contribute to permanently increased oxidative stress or damage [42]. This, in combination with the under-production of antioxidant defensive mechanisms, can lead to obesity-related complications, co-morbidities and increased risk factors for NCDs [28,42]. Therefore, beneficial changes to body composition may improve health and overall quality of life, and increasing evidence supports dietary flavonoids as beneficial for weight maintenance and biomarkers relating to body composition [43,44]. A study of 2734 female participants found that a higher intake of foods naturally rich in ACNs and flavone subclasses was inversely associated with significantly lower fat mass and abdominal fat accumulation [43]. Additionally, Takahashi et al. (2015) examined the consumption of chokeberries in rats, in which visceral fat accumulation was suppressed, potentially via inhibiting pancreatic lipase activity [45]. In an obese mouse model, freeze-dried blueberry and strawberry powders were assessed in relation to weight gain and body fat accumulation as a combination of a high- or low-fat diet, or as standalone supplements [46]. The standalone supplements proved to be more effective at inhibiting weight gain and the accumulation of body fat, as opposed to consuming them with or as part of a meal [46]. These findings are useful in determining the pharmacokinetics of particular ACN derivatives, and further clinical studies are needed to identify any additional potential benefits. Overall, the management and maintenance of body composition through ACN consumption is an attribute that can have beneficial ‘flow-on’ effects for the prevention and mitigation of chronic diseases.

### 3.3. Chronic Inflammation and Metabolic Syndrome

Chronic inflammation has an extensive role in the pathogenesis of obesity and other associated metabolic diseases including insulin resistance, type 2 diabetes mellitus (T2DM) and CVD [34,41]. The potential to target inflammation is thus an appealing strategy to combat inflammatory-related diseases [32,34]. Ameliorating this burden with food-derived products, or ‘bioactives’, is a relatively novel alternative to a traditional pharmaceutical approach; however, the subsequent alteration of metabolic markers is a common goal that, with limited side effects, is an efficient and cost-effective strategy worthy of consideration [3,32]. Among other pathways, obesity influences the activation of the innate immune system in adipose tissue that promotes pro-inflammatory pathways and induces ROS/RNS production, triggering a systemic acute immune response [42]. It is believed that impaired ability of adipocytes storing excess energy as triglycerides contributes to the accumulation of lipids and their metabolites in other tissues that are not necessarily adapted to lipid storage [42]. Inevitably, increased adiposity leads to oxidative stress, inducing monocyte infiltration into adipose tissue, and this innate immune response increases the risk for atherosclerotic plaque development, and ultimately, CVD [42,47]. A study by Kim et al. (2016) showed that blackcurrants (prepared from a dried powder, 6.3% *w/w*) inhibit macrophage infiltration in adipose tissue of obese mice, which is a vast contributor to the development of metabolic disorders via cytokine secretion by macrophages in adipose tissue—these cytokines play a role in promoting insulin resistance [48]. Insulin resistance occurs early in the progression of metabolic disorders and plays a pivotal role in the pathogenesis of T2DM. Human studies have suggested that ACNs could improve glucose tolerance, insulin sensitivity and reduce inflammatory markers such as Interleukin-6 (IL-6) through several cellular and molecular mechanisms [34,42]. Furthermore, the findings of a study by Vugic et al. (2020) in lean, overweight and obese individuals have also indicated that 320 mg/day of total ACNs decreased levels of IL-6 in the obese treatment group [36]. However, there is a lack of consensus regarding the effects of ACN consumption on insulin resistance. Homeostatic Model Assessment of Insulin Resistance (HOMA-IR) was significantly altered after six weeks of supplementation with tart cherry juice in diabetic women—suggesting that weight reduction and increased insulin sensitivity may be attainable through supplementation with ACN-rich juices [49]. In contrast, a 2019 study in 28 healthy participants supplementing with tart montmorency cherry for four weeks did not alter HOMA-IR or IL-6 [50]. Despite non-significant results of HOMA-IR in healthy populations, HOMA-IR was significantly reduced in diabetic, pre-diabetic and obese and overweight populations [51]. Purple potato extracts containing acylated ACNs have shown reductions in postprandial blood glucose and insulin peaks in an acute, randomized cross-over trial, indicating that this may alleviate postprandial glycemia and insulinemia [52]. In addition to the proposed metabolic benefits of ACNs, their anti-inflammatory properties have also shown beneficial results in pain reduction and in inflammation in human clinical trials for inflammatory arthritis, which is often associated with obesity in later life, in addition to the increased risk of CVD [53].

### 3.4. Cardiovascular Disease

The consumption of foods high in ACN content may contribute to positive cardiovascular effects [54]. Several systematic reviews and meta-analyses have investigated the effects of ACNs on cardiometabolic health, including factors such as lipid profile and hypertension [27,51,54,55]. Throughout CVD development, oxidative stress causes vascular inflammation, upregulating lipid molecules and thereby unfavourably altering levels of high-density lipoprotein cholesterol (HDL-C) and low-density lipoprotein cholesterol (LDL-C) [51,55]. This, in turn, results in the upregulation of cell adhesion molecules and increases the risk for vascular adversity [51]. As potential cardioprotective agents, ACNs have also been shown to attenuate oxidized LDL-C (oxLDL)-mediated foam cell formation—one of the main drivers of atherogenesis—through inflammatory pathways, and more recently involving the regulation of CD36 gene expression (responsible for the encoding of glycoprotein IV) [56]. For example, ACN-rich hibiscus extract has prevented lipid accumulation, decrease CD36 expression and inhibit the macrophage uptake of oxLDL *in vitro* [56]. Additionally, results obtained from a double-blind, randomized, placebo-controlled trial showed that the consumption of a berry-derived ACN supplement (160 mg twice daily) for 12 weeks significantly increased serum HDL-C and decreased LDL-C when compared with placebo [57]. A study by Asgary et al. (2014) evaluated the cardiometabolic effects of pomegranate juice (150 mL/day) for two weeks in hypertensive patients aged between 30 and 37 years [58]. Results from this trial indicated that pomegranate juice significantly reduced diastolic and systolic blood pressure, but not flow-mediated dilation [58]. As CVD, and associated risk markers, have both modifiable lifestyle and inherited factors, populations considered to be at increased risk are overweight and obese populations, smokers, those with a family history, and postmenopausal women, specifically. Supplementing with elderberry extract (500 mg/day) for 12 weeks was shown to be ineffective in reducing risk of CVD in healthy, postmenopausal women [59]. This result was intriguing, as postmenopausal status is commonly associated with an increase in oxidative stress [19]. Given the study length, and that estrogen has shown a potential cardioprotective benefit in females, it may be worth exploring the concept of ACN supplementation in the presence and absence of particular sex hormones, as this may inhibit the efficacy of its scavenging effects in relation to cardiovascular health [19,59]. However, bilberries were shown to be effective in improving bone health in menopause populations [19] and a human clinical trial conducted in 2016 assessing bilberry supplementation reported significantly reduced CVD risk factors, by inducing favorable changes in the lipoprotein profiles in menopausal women [60]. This may be more of a reflection of the type of ACN food constituents, rather than the efficacy of ACNs in general. The overall findings indicate that ACN consumption may have beneficial effects on cardiometabolic biomarkers, and support their consideration as an alternative method of prevention and treatment of CVD and associated risk markers. Further long-term trials are needed to explore optimal ACN food sources, dosage and duration in a range of at-risk populations.

### 3.5. Alzheimer’s, Parkinson’s and Other Neurological Diseases

Approximately 20% of the bodies total oxygen supply is consumed by the brain, of which, a significant portion is converted to ROS [29]. As oxidative damage poses stress on DNA, proteins and lipids, the brain is vulnerable to neuronal degradation and can progress to neurological pathology such as Alzheimer’s Disease (AD) and Parkinson’s Disease (PD) [23]. The pathogenesis of AD is considered to have a multifaceted origin and oxidative stress has been widely accepted in its etiology, as well as consideration of lifestyle factors including poor diet and sedentary behavior, both of which have been shown to accelerate Amyloid beta (Aβ) accumulation [29,61,62]. Similarly, RNS are also found to contribute to AD pathology through excitotoxicity of N-methyl-D-aspartate receptors, where they become permeable to calcium, potassium and sodium ions [61,63]. Abnormal intracellular ion levels induce mitochondrial damage and ROS production by increasing the mitochondrial ion burden and inducing mitochondrial dysfunction [24,63]. On the other hand, PD is characterized by a loss of dopaminergic neurons [64], and while the exact etiology of PD remains unclear, the understanding of oxidative stress in relation to dopamine metabolism and neuroinflammation remain important informants in its progression [23,64]. The ACNs were also investigated for their ability to diminish glutamate-induced neurotoxicity, and their antioxidant activities are of great interest in AD and PD [23]. The in vitro study using the purple sweet potato showed a strong free radical scavenging effect and reduced Aβ-induced-toxicity in an animal derived PC12 cell line [20]. Additionally, mulberry fruit isolates have also been reported to have some degree of neuroprotection against cerebral ischemia in a PC12 cell line, indicating the capacity to minimize ischemic cell death as a result of vascular narrowing or stroke [65]. This could potentially improve brain-trauma outcomes and overall quality of life in stroke patients. The neuroprotective benefits of blueberry juice has also shown promise in enhancing signalling pathways and preventing behavioural deficits in an AD mouse model, and a recent human trial conducted by Krikorian et al. (2020) highlighted that this may be a possible path to overcome genetic predisposition to AD [66,67]. However, there is only mixed, preliminary evidence supporting the use of blueberries and blueberry products on cognitive performance and mood [68]. There remains a need for future consideration of ACN derivatives as a novel therapeutic approach to managing the progression of life-changing neurological illnesses.

### 3.6. Delayed Onset of Disease

As ACNs and their metabolites have shown promise as potential therapeutic agents for neurodegenerative diseases such as AD and PD, further investigation of prolonged healthspan in individuals predisposed to, or with preexisting neuropathologies, is important [23]. Amyotrophic Lateral Sclerosis (ALS), or more socially termed ‘Lou Gehrig’s Disease’, is currently an incurable degenerative disorder affecting the central nervous system (CNS) and eventually leads to diminishing skeletal muscle integrity [69]. Pre-symptomatic supplementation with ACN-enriched extract from strawberries significantly delayed disease onset in an ALS mouse model [70]. The study reported a delay in disease onset, in addition to a significant extension in survival rates in this animal model [70]. These findings provide support for future exploration in human trials, as well as potential preclinical models of other neurodegenerative diseases. Aging has been associated with an increase in DNA damage, and without effective response and repair pathways, proliferating stressors can lead to irreparable DNA damage [71]. The aging of the liver is a natural process that occurs at a stable but irreversible rate; therefore, a decline in liver function occurs concurrently with the aging process [72]. Age-related liver diseases are accompanied by high rates of morbidity and mortality, increasing disease risk such as chronic hepatitis and liver cirrhosis [72]. In recent years, the black chokeberry has been widely used due to its high ACN content and relatively strong antioxidant activity [33]. A study by Wei et al. (2017) observed the effects of black chokeberry supplementation (eight weeks) on DNA damage inhibition and liver function in mice [33]. Findings indicated that the treatment groups were more effective in slowing the age-related degeneration of the whole liver, highlighting that ACNs could be a promising and accessible potential source of anti-aging agents.

### 3.7. Emerging Evidence

**Figure 2:**
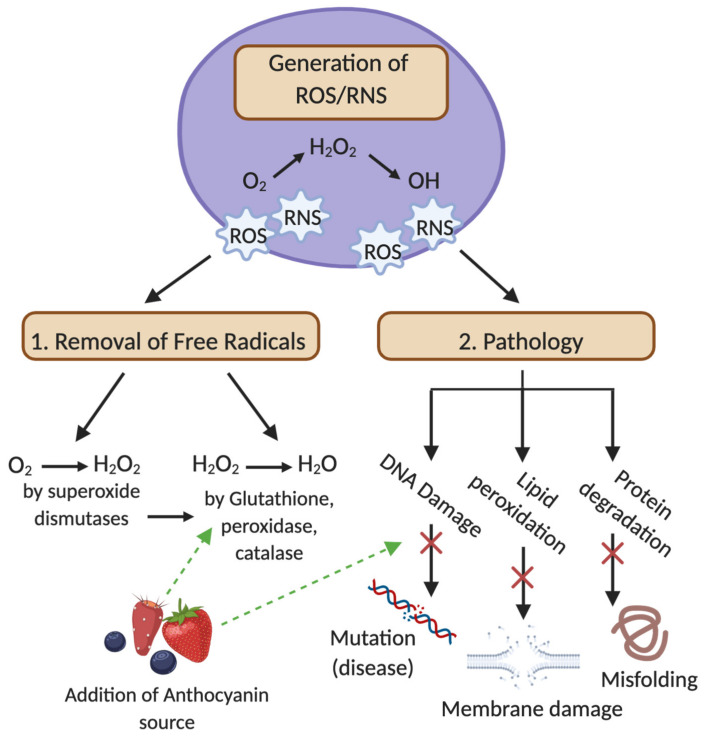
A proposed pathway facilitating the removal of ROS/RNS by internal antioxidant systems with ACN support. ROS/RNS production is a natural process occurring in most aerobic cells. Increased production is therefore related to injurious stimuli. Internal antioxidant systems are present to facilitate the removal of normal ROS/RNS production (pictured at point 1); however, inadequate removal or excessive production can result in cellular pathology such as lipid, protein and DNA damage (pictured at point 2). Anthocyanins can support this facilitated removal through their additional antioxidant properties, preventing further pathological progression.

There are several health-promoting benefits attributable to the inhibition of oxidative stress proposed by ACN consumption (Figure 2), including the modulation of gut microbiota [73]. Some dietary ACNs have been studied for their ability to act as prebiotics, beneficially modulating the gut microbiome [74]. The changes in gut microbiota have also been strongly associated with obesity, and recent studies have shown that non-absorbable flavonoids or polyphenol subclasses alter the gut microbial community, resulting in lower systemic inflammation and improved metabolic outcomes [26,74,75]. Interestingly, there is emerging evidence suggesting that the polyphenols and ACNs present in red wine pose beneficial effects to the gut microbiota not observed in other alcoholic beverages [26,76]. Together, these are novel leads in the potential of non-absorbed compounds in treating metabolic disorders. In addition to their cardioprotective and anti-inflammatory properties, the intake of berries may improve bone health, and often overlooked are the effects that ACNs could have on populations who are increasingly susceptible to CVD and other inflammatory diseases such as osteoporosis and arthritis. Relatively recently, it has been indicated that the endocrine system is involved in the modulation of oxidative stress through hormonal homeostasis [19]. Oxidative stress is evident in the pathogenesis of many endocrine diseases as well as bone degeneration [19,77]. Polyphenols, as a broader class, have been shown to protect bone health through the modulation of osteoblastogenesis, osteoclastogenesis and osteoimmunological action [78]. Importantly, this emerging research on bone health paves the way for the general use of ACNs as therapeutic interventions for individuals suffering from inflammatory, menopausal or age-related bone degeneration.

Segments of DNA such as telomeres are highly sensitive to oxidative damage, and the diet may impact telomere attrition rates with respects to mediating oxidative stress and inflammation. Telomeres are nucleoprotein complexes capping the ends of chromosomes, serving to protect eukaryotes from DNA damage, preventing end-to-end fusion and limiting the capacity for extensive cellular replication [79,80]. Telomeres naturally shorten with every round of DNA replication, eventually leading to cellular senescence [80]. Increased telomere attrition rates are associated with reduced relative telomere length and genomic instability—increasing the risk of DNA damage, excessive production of ROS/RNS and linking to the progression and development of NCDs and reduced longevity [79,80]. Recently, it has been proposed that consumption of food rich in ACNs, through their antioxidant properties complementing the removal of excessive ROS/RNS, may relieve the extensive damage caused to DNA as a result of oxidative stress [81]. This may delay shortening of telomeres, decrease the risk of NCDs and promote longevity—an ultimate goal encouraging ‘food for thought’.

## 4. Summary of Findings

An important element to recognize throughout this review is that there are several overlapping and ‘flow-on’ effects that influence individual physiological systems and human health as a whole. Oxidative stress is a key player in disease onset and progression, and ACNs consumption may mitigate this by counteracting the oxidative damage to biological molecules such as lipids and DNA. Although inflammation is regarded as a normal physiological response, when considered from a pathological perspective or as a chronic condition, it can pose extensive stress on the body. Both inflammation and oxidative stress have the potential to be offset with the anti-inflammatory and antioxidant properties of ACNs. The most common sources of ACNs explored in this review were predominantly from berries (blueberry, strawberry, mulberry, chokeberry, bilberry, and elderberry). Importantly, the consumption and availability of specific ACNs differ broadly among demographic populations, geographical location, as well as with climate and seasons. Therefore, daily consumption or recommended consumption to see expected results remains controversial. Generally, study designs that used a longer intervention period (greater than 12 weeks) observed positive effects on cardiometabolic factors, body composition and suggest ACNs may be effective in reducing HOMA-IR in obese adults. Delayed onset of disease in animal models is also one of the key takeaways from this review, with which timely and appropriate ACN supplementation has the potential to increase healthspan and overall quality of life.

Overall, the findings from this review indicate that ACNs exhibit antiatherogenic, antioxidant, anti-inflammatory and anti-apoptotic properties. However, their composition differs vastly in many food sources, and thus potentially in the physiological effects after consumption.

## 5. Conclusions and Future Directions

We reviewed the effects of ACNs beyond their antioxidant capacity in relation to overall human health, specifically regarding NCD progression and prevention. Sources of ACNs found naturally in the diet including, but not limited to strawberries, mulberries, blueberries, elderberry and pomegranate, may represent a suitable non-pharmaceutical approach to the regulation of oxidative stress, and inflammatory-related diseases. A vast majority of the literature included were from animal models or in vitro studies. While promising, the findings from these studies warrant further exploration in human populations. This review did not focus on the bioavailability of specific ACNs or ACN-rich food sources, and with a plethora of literature available it is recommended to consider this information when designing human trials, as there are factors affecting absorption that may render the molecules less effective.

Overall, consumption of ACNs as standalone or combination therapy may prove to be a reliable and effective approach to the prevention and management of age or inflammatory-related diseases.

## Figures and Tables

**Table 1:** Range of total anthocyanin content in commonly consumed fruits ^1^.

Fruit	Total Content (mg/kg)
Bilberry	4600
Blackberry	820–1800
Blueberry	825–5300
Cherry	3500–4500
Chokeberry	5060–10000
Elderberry	2000–15600
Raspberry	100–600
Strawberry	127–360

^1^ Table adapted from Horbowicz et al. (2008) [12].
